# Differentiated thyroid carcinoma: what the nonspecialists needs to know

**DOI:** 10.20945/2359-4292-2023-0375

**Published:** 2024-02-29

**Authors:** Ana O. Hoff, Aline Lauda Freitas Chaves, Thiago Bueno de Oliveira, Helton Estrela Ramos, Gustavo Cancela Penna, Lucas Vieira dos Santos, Ana Luiza Maia, Daniel Oliveira Brito, Franco Pelissari Vizzotto

**Affiliations:** 1 Faculdade de Medicina da Universidade de São Paulo, São Paulo Instituto do Câncer do Estado de São Paulo Disciplina de Endocrinologia e Metabologia São Paulo SP Brasil Disciplina de Endocrinologia e Metabologia, Instituto do Câncer do Estado de São Paulo, Faculdade de Medicina da Universidade de São Paulo, São Paulo, SP, Brasil; 2 DOM Oncologia Divinópolis MG Brasil DOM Oncologia, Divinópolis, MG, Brasil; 3 A.C.Camargo Cancer Center São Paulo SP Brasil A.C.Camargo Cancer Center, São Paulo, SP, Brasil; 4 Universidade Federal da Bahia Departamento de Biorregulação Instituto de Saúde e Ciências Salvador BA Brasil Departamento de Biorregulação, Instituto de Saúde e Ciências, Universidade Federal da Bahia, Salvador, BA, Brasil; 5 Universidade Federal de Minas Gerais Departamento de Clínica Médica Faculdade de Medicina Belo Horizonte MG Brasil Departamento de Clínica Médica, Faculdade de Medicina, Universidade Federal de Minas Gerais, Belo Horizonte, MG, Brasil; 6 Unidade de Câncer de Cabeça e Pescoço Hospital Beneficência Portuguesa de São Paulo São Paulo SP Brasil Unidade de Câncer de Cabeça e Pescoço, Hospital Beneficência Portuguesa de São Paulo, São Paulo, SP, Brasil; 7 Universidade Federal do Rio Grande do Sul Hospital de Clínicas de Porto Alegre Porto Alegre RS Brasil Hospital de Clínicas de Porto Alegre, Universidade Federal do Rio Grande do Sul, Porto Alegre, RS, Brasil; 8 Oncoclínicas Bahia – Núcleo de Oncologia da Bahia Salvador BA Brasil Oncoclínicas Bahia – Núcleo de Oncologia da Bahia, Salvador, BA, Brasil; 9 United Medical Ltda. (Knight Therapeutics) São Paulo SP Brasil United Medical Ltda. (Knight Therapeutics), São Paulo, SP, Brasil

**Keywords:** Thyroid cancer, differentiated thyroid carcinoma, kinase inhibitor, lenvatinib, sorafenib

## Abstract

Differentiated thyroid carcinoma (DTC) accounts for most cases of thyroid cancer, and the heterogeneity of DTC requires that management decisions be taken by a multidisciplinary team involving endocrinologists, head and neck surgeons, nuclear medicine physicians, pathologists, radiologists, radiation oncologists, and medical oncologists. It is important for nonspecialists to recognize and refer patients with DTC who will benefit from a specialized approach. Recent advances in knowledge and changes in management of DTC call for the need to raise awareness on the part of these nonspecialist physicians, including general endocrinologists and medical oncologists at large. We provide an overview of diagnostic and therapeutic principles in DTC, especially those that bear direct implication on day-to-day management of these patients by generalists. Patients with DTC may be broadly categorized as having localized, locally persistent/recurrent, or metastatic disease. Current recommendations for DTC include a three-tiered system that classifies patients with localized disease into low, intermediate, or high risk of persistent or recurrent disease. Risk stratification should be performed at baseline and repeated on an ongoing basis, depending on clinical evolution. One of the overarching goals in the management of DTC is the need to personalize treatment by tailoring its modality and intensity according to ongoing prognostic stratification, evolving knowledge about the disease, and patient characteristics and preference. In metastatic disease that is refractory to radioactive iodine, thyroid tumors are being reclassified into molecular subtypes that better reflect their biological properties and for which molecular alterations can be targeted with specific agents.

## INTRODUCTION

The complexity and heterogeneity of thyroid cancer require that management decisions be taken by a multidisciplinary team. Specialists involved in thyroid cancer are endocrinologists, head and neck surgeons, nuclear medicine physicians, pathologists, radiologists, radiation oncologists, and medical oncologists. However, it is important for nonspecialists to recognize and refer patients who will benefit from a specialized approach. Recent advances in knowledge and changes in management of thyroid cancer call for the need to raise awareness on the part of generalist physicians, including endocrinologists and medical oncologists not specialized in thyroid cancer. This is particularly important in Brazil and other countries characterized by disparities in access to state-of-the-art medical care. This article aims to provide generalists with an overview of differentiated thyroid carcinoma (DTC), which accounts for most cases of thyroid cancer.

## EPIDEMIOLOGY AND CLASSIFICATION

The epidemiology of thyroid cancer is characterized by a disconnect between incidence and mortality. Worldwide, 586,202 new cases of thyroid cancer were estimated in 2020; the estimated number of deaths for that same year was 43,646 (1). This low case-fatality rate, mostly due to the indolent nature of DTC, is another reason to emphasize the importance of adequate patient management, because the relatively rare patients with aggressive disease must be recognized and treated accordingly, whereas their lower-risk counterparts require a more conservative approach. A second explanation for the low case-fatality rate of thyroid cancer is the fact that a large proportion of cases diagnosed through ultrasound screening would not have clinical consequence, especially when it comes to papillary tumors measuring less than 1-2 cm. This "incidental" finding on screening is corroborated by the frequent finding of thyroid cancer in autopsies of persons dying from other causes (2). Therefore, after decades of increase in the US, incidence rates of DTC have declined since 2014, largely because of decreased overdetection (3,4). Conversely, in South Korea, where screening for thyroid cancer has become widespread, the rate of DTC diagnosis in 2011 was 15 times that observed in 1993, with no change in mortality (5). Qualitatively similar findings have been reported in Brazil, where screening for thyroid cancer is not recommended at the national level (6,7). Thyroid cancer is more frequent in women, who accounted for 448,915 of the expected new cases worldwide in 2020 (1), 31,180 of the total of 43,720 in the US in 2020 (3), and 11,950 of the total of 13,780 in Brazil in 2020 (8). As a result, thyroid cancer is the fifth most frequent tumor type among women worldwide and in Brazil, and the seventh in the US (1,3,8). The mean age at diagnosis for patients with DTC is approximately 45 years (9,10).

The World Health Organization classification of thyroid tumors was recently updated (11). Table 1 displays salient features of the most frequent histological types of DTC (12-15). Papillary carcinoma, follicular carcinoma, and oncocytic carcinoma (previously known as Hürthle-cell carcinoma), collectively denominated DTC, constitute between 90% and 95% of cases of thyroid cancer and are the sole focus of this review (13,14). Medullary and anaplastic thyroid carcinomas, although less frequent, deserve special consideration and are discussed in recent reviews (16,17). Papillary carcinoma is the most frequent type of thyroid cancer; despite molecular and clinical heterogeneity, papillary carcinomas have a better overall prognosis than follicular tumors (13,14).

**Table 1:** Selected features of the most frequent histological types of differentiated thyroid carcinoma (10-15,20,23,25)

Features	Papillary carcinoma	Follicular variant of papillary carcinoma	Follicular carcinoma	Oncocytic carcinoma
Relative frequency*	75%-80%	3%-5%	2%-5%	~2%
Histological subtypes	Now classified, irrespective of tumor size, as classic, infiltrative follicular variant, diffuse sclerosing, Warthin-like, solid, oncocytic papillary, tall-cell, columnar-cell, and hobnail subtypes.	Comprises infiltrative and encapsulated subtypes.	Invasive but lacking nuclear cytology of papillary tumors. Comprises minimally invasive, encapsulated angioinvasive, and widely invasive subtypes.	Previously called Hürthle-cell carcinoma, its classification requires histological evidence of malignancy and absence of high-grade features. Comprises minimally invasive, encapsulated angioinvasive, and widely invasive subtypes.
Molecular landscape	*BRAF* mutations in ~60%, *TERT* mutations (from 9% in some series to up to 61% of very advanced cases), *RAS* mutations in ~10%, *RET* fusions in ~5% of cases, and *NTRK* fusions in 1%-3%.	*RAS* alterations in ~30%, *BRAF* mutations in 20%.	*RAS* mutations in 66% of advanced cases, and *TERT* mutations (in up to 71% of very advanced cases). Also, alterations in *PTEN* and *RB1*.	Alterations in mitochondrial genes and duplications of chromosomes 5 and 7 are key genomic alterations. Notable absence of *BRAF* mutations, only 10% with *RAS* mutations. *TERT* promoter mutations in ~60%.
Pattern of spread and clinical behavior	Lymph-node metastasis at diagnosis in ~25%. Lung as only site of metastasis more frequent than in follicular carcinoma. Bone metastasis less frequent than in follicular carcinoma. Overall, better prognosis than follicular carcinoma. *BRAF* V600E mutation associated with more aggressive disease and RAI refractoriness.	Unlikely to recur or metastasize.	Lung as only site of metastasis less frequent than in papillary carcinoma. Bone metastasis more frequent than in papillary carcinoma.	Widely invasive subtype is characterized by extensive capsular and vascular invasion, frequent lung and bone metastases, and RAI refractoriness.

* Considering all subtypes of thyroid cancer. Abbreviation: RAI, radioactive iodine.

### Molecular pathology and therapeutic implications

Findings from DNA sequencing studies are increasingly revealing the genetic basis for thyroid cancer (18,19). In consequence, thyroid tumors are being reclassified into molecular subtypes that better reflect their biological and clinical features. Moreover, targetable molecular alterations are being explored to improve patient management (14,20,21). Even though genetic testing is not mandatory for most patients with DTC, it is beneficial for therapeutic decisions and may have prognostic importance in locally advanced or distant metastatic disease (22). Most thyroid tumors harbor mutations in members of the mitogen-activated protein kinase (MAPK) signaling pathway, which transmits signals from the plasma membrane to the nucleus (20). These mutations often lead to activation of the proteins they code, thus leading to increased tumor-cell proliferation. Two protein kinases in the MAPK pathway are key players in DTC: *BRAF* and *RAS*. Interestingly, these driver mutations – *i.e.*, mutations with a prominent pathogenic role – are associated with different, and often mutually exclusive, tumor entities (Table 1). For example, *BRAF* pathogenic variants (typically, the V600E mutation) are present in 60%-70% of papillary tumors, which have *RAS* mutations in approximately 10%-15% of cases and *RET* alterations in nearly 5%-7% of cases (although they seem to be more frequent in the pediatric population) (13,18,23,24). Although less is known about follicular tumors, studies have shown frequent (nearly 45%) *RAS* mutations and mutations in the telomerase reverse transcriptase (*TERT*) gene promoter (23). Of note, *TERT*-promoter mutations have been found in nearly 10% of papillary tumors, in which they seem to be associated with a high risk of recurrence (18). Likewise, the molecular profiles of follicular variant papillary tumors and oncocytic tumors have been characterized to some extent (Table 1) (20,23,25). Genetic abnormalities can be assessed in clinical practice using various next-generation sequencing (NGS, which can assess both DNA and RNA) platforms and, increasingly, liquid biopsy (*i.e.*, detection of circulating tumor DNA); however, access to molecular testing may be problematic in some health care settings. Depending on access to testing, an alternative strategy to NGS testing is a multistep approach, with initial testing for *BRAF* followed by subsequent testing for other alterations individually or collectively (26).

Over the past two decades, systemic treatment for cancer has progressively migrated from conventional chemotherapy to targeted therapy and immunotherapy. Although the latter is still incipient in thyroid cancer, targeted therapy has become the mainstay of treatment for various subtypes of advanced DTC (13,14,20). In fact, thyroid cancer has been considered the malignancy with the second highest rate of targetable driver mutations (19). Selective inhibitors of tyrosine or serine-threonine kinases are oral drugs that block various signaling pathways, among which the MAPK pathway is the most relevant in DTC. Moreover, some kinase inhibitors have antiangiogenic properties due to inhibition of different vascular endothelial growth factor (VEGF) receptors, which are located upstream of the MAPK pathway. Other agents target very specific kinases – such as neurotrophic receptor tyrosine kinase (NTRK) and rearranged during transfection (*RET*) – that drive specific tumor types. Table 2 provides a summary of the most important kinase inhibitors used in the current management of DTC and approved by the US Food and Drug Administration (FDA) (27,32). These agents, generally indicated only for patients with radioactive iodine (RAI)-refractory thyroid cancer, are discussed in greater detail in the section *Overview of Systemic Treatment*. Of note, only cabozantinib, larotrectinib, lenvatinib and sorafenib are commercially available in Brazil at the time of writing, regardless of specific approval in DTC or current reimbursement practices.

**Table 2:** Selected features of kinase inhibitors used in differentiated thyroid cancer (27-32)

Features	Cabozantinib	Larotrectinib	Lenvatinib	Pralsetinib	Selpercatinib	Sorafenib
Target(s)	VEGF receptor 2, KIT, FLT-3, *RET*, MET	NTRK	VEGF receptors 1, 2, and 3, FGF receptors 1 to 4, PDGF receptor, KIT, and *RET*	*RET*, VEGF receptor 2	*RET*, VEGF receptor 2	VEGF receptors 1, 2, and 3, PDGF receptor, FGF receptor, KIT, and *RET* (weak BRAF inhibitor)
Approval by the US FDA	Locally advanced or metastatic differentiated thyroid cancer, progressing after VEGF receptor-targeted therapy and ^131^I-refractory or ineligible	Metastatic or unresectable tumors with NTRK fusion, no acquired-resistance mutation, and no satisfactory alternatives	Locally recurrent or metastatic, progressive, ^131^I-refractory differentiated thyroid cancer	Advanced or metastatic *RET* fusion-positive ^131^I-refractory thyroid cancer	Advanced or metastatic *RET* fusion-positive ^131^I-refractory thyroid cancer	Locally recurrent or metastatic, progressive, ^131^I-refractory differentiated thyroid cancer
Capsule/tablet strengths	20 mg, 40 mg, and 60 mg	25 mg and 100 mg (also, solution with 20 mg/mL)	4 mg and 10 mg	100 mg	40 mg and 80 mg	200 mg
Most frequent adverse events in pivotal trials	Diarrhea, fatigue, hand-foot syndrome, decreased appetite, hypertension, nausea, vomiting, weight loss, and constipation.	Fatigue, nausea, dizziness, vomiting, anemia, increased transaminases, constipation, and diarrhea.	Hypertension, diarrhea, fatigue or asthenia, and nausea.	Constipation, hypertension, fatigue, musculoskeletal pain, and diarrhea.	Dry mouth, diarrhea, hypertension, fatigue, edema, rash, and constipation.	Hand-foot syndrome, diarrhea, alopecia, rash, fatigue, and hypertension.

Abbreviations: FDA, Food and Drug Administration; FGF, fibroblast growth factor; FLT-3, Fms-related receptor tyrosine kinase 3; MET, mesenchymal-epithelial transition; NTRK, neurotrophic receptor tyrosine kinase; PDGF, platelet-derived growth factor; RET, rearranged during transfection; VEGF, vascular endothelial growth factor.

### General principles of patient management

#### Multidisciplinary approach

An overarching principle in the management of patients with DTC is the need for a multidisciplinary approach (22,33-35). Even though each specialist plays a larger role in specific treatment phases, all specialists benefit from multidisciplinary discussions and knowledge exchange among specialties. Given the long natural history and heterogeneous behavior of DTC, patients likewise benefit from such collaboration. Tumor boards are ideal for discussion and should be implemented whenever possible. Likewise, referral to specialist centers is paramount in the attempt to optimize results (36,37).

#### Staging principles and risk stratification

Patients with DTC may be broadly categorized as having localized, locally persistent/recurrent, or metastatic disease. Most patients have localized disease at diagnosis; even though the majority will be cured, approximately 20% of patients are destined to transition to one or both of the other two states. Overall, distant metastases occur in up to 10% of patients with DTC; in these patients, metastases may be found decades after initial treatment, but 77% of local or distant recurrences are discovered during the first 5 years after surgery (10,13,33). In a large series of 444 patients with distant metastases from papillary or follicular tumors treated between 1953 and 1994, 50% had lung metastases only, 26% had bone metastases only, 18% had both lung and bone metastases, and 5% had metastases at other sites (15).

The 8^th^ edition of the American Joint Committee on Cancer (AJCC)/Tumor-Node-Metastasis (TNM) classification is currently used to stage patients with DTC (38,39). In addition to the usual clinical and pathological features accounted for in the AJCC/TNM system, age (now 55 years and above, unlike the previous 45-year cutoff) is an important prognostic factor in DTC. Thus, patients younger than 55 years have a better outlook and are only grouped between stages I and II, whereas older patients are grouped in stages I to IV. The expected 10-year disease-specific survival is 98%-100% for stage I, 85%-95% for stage II, 60%-70% for stage III, and less than 50% for stage IV (39). While the AJCC/TNM classification is prognostic for mortality, the American Thyroid Association (ATA) has created a classification system to predict the risk of recurrence. The ATA Risk Stratification System classifies patients into low (≤5%), intermediate (6%-20%), or high (>20%) risk of persistent or recurrent disease (40). Low risk is defined as intrathyroidal DTC with clinically negative or up to five pathologically positive lymph-node micrometastases (<0.2 cm); intermediate risk, as any of microscopic invasion into the perithyroidal soft tissues, RAI-avid metastatic foci in the neck on the first posttreatment whole-body scintigraphy, aggressive histology, papillary tumor with vascular invasion, clinically positive lymph nodes, more than five pathologically positive lymph nodes (<3 cm), or multifocal papillary microcarcinoma with extrathyroidal extension and *BRAF* V600E mutation; and high risk, as any of incomplete tumor resection, gross extrathyroidal extension, pathologically positive lymph nodes with any ≥3 cm, follicular tumor with extensive vascular invasion (more than four foci of vascular invasion), postoperative thyroglobulin suggestive of distant metastases, or presence of distant metastases.

Risk stratification should be performed at baseline, as it can inform therapeutic and early follow-up decisions; however, it should be repeated on an ongoing basis, depending on clinical evolution. Therefore, a system that incorporates individual response to therapy into a real-time, dynamic risk stratification scheme is proposed by ATA (40). For example, patients initially classified as intermediate- or high-risk can be reclassified as having low risk of recurrence based on excellent responses to initial therapy. For this, all clinical, biochemical, imaging, and pathological findings during follow-up are used to redefine the clinical status and assess response to therapy (40,41). At the very least, an assessment is recommended 6-18 months after primary surgical treatment (± RAI therapy) for localized disease (13,22,33). Four categories of response are recognized: (1) excellent response, when the patient has no clinical, biochemical, or structural (*i.e.*, anatomical) evidence of disease; (2) biochemical incomplete response, when the patient has abnormal thyroglobulin or rising antithyroglobulin antibodies in the absence of structural disease; (3) structural incomplete response, when the patient has persistent or newly identified locoregional or distant metastases; and (4) indeterminate response, when the patient has nonspecific biochemical or structural findings that cannot be classified as either benign or malignant (including stable or declining antithyroglobulin antibodies without definitive structural disease).

#### Biochemical markers

Thyroglobulin, synthesized by thyroid follicular cells in response to thyroid-stimulating hormone (TSH), plays a key role in the synthesis of thyroid hormones, *i.e.*, triiodothyronine (T3) and thyroxine (T4) (42). Thyroglobulin is the primary tumor marker in DTC and plays a key role in prognostic assessment and follow-up, except in the 5%-10% of patients with undetectable levels of this marker. With a half-life between 1 and 3 days, a postoperative nadir in serum thyroglobulin level is expected 3-4 weeks after surgery. Serum thyroglobulin level can be measured by radioimmunoassay, immunometric assays, and liquid chromatography-tandem mass spectrometry. Immunometric assays are widely available, and ideally, patient follow-up should be performed in the same laboratory using the same assay each time. Antithyroglobulin antibodies, present in approximately 25% of patients with thyroid cancer and 10% of the general population, are polyclonal autoantibodies that falsely lower thyroglobulin levels in immunometric assays. Therefore, antithyroglobulin antibodies should be measured in conjunction with thyroglobulin for proper interpretation (33,34). Both thyroglobulin and antithyroglobulin antibodies are integral components of the ATA response categories (40). Declining antithyroglobulin antibodies are a good prognostic sign, while rising levels or first appearance of these antibodies are associated with persistent or recurrent disease (33,42). If RAI is planned (see below), thyroglobulin and antithyroglobulin antibodies should be measured before administration (34).

#### Imaging methods

Neck ultrasound plays a key role in the diagnosis and follow-up of thyroid cancer; it provides information on tumor size and location, number of lesions, lymph nodes, and macroscopic invasion of surrounding tissues (13). A second imaging method in DTC is whole-body scintigraphy, which may be used after surgery to assess its completeness (using 123I or low doses of 131I, the former being less widely available) or after therapeutic RAI administration (22,40). However, the role of whole-body diagnostic scintigraphy in routine practice is controversial; the method has low sensitivity and is not indicated during follow-up (22,33,40). Other imaging modalities are required for initial assessment only if neck ultrasound provides doubtful results, or locoregional or distant metastases are suspected (33,34,40). Computed tomography (CT) scan of the neck and chest, or magnetic resonance imaging for patients unsuitable for CT scans, is useful in the assessment of structural disease. Positron-emission tomography (PET) and PET-CT are increasingly used and allow for whole-body assessment, including bones, and are particularly useful in aggressive variants of papillary tumors (such as tall-cell and hobnail), poorly-differentiated disease, rising thyroglobulin in a context of stable structural disease by other imaging studies, and in RAI-refractory disease (22,33,34). On the other hand, bone scans are not very useful, as bone metastases in DTC are usually lytic rather than blastic (43,44).

### Treatment personalization

A final overarching goal in the management of thyroid cancer is the need to personalize treatment; this can be achieved by tailoring treatment modality and intensity according to ongoing prognostic stratification, evolving knowledge about the disease, and patient characteristics and preference (34,40,45).

### Overview of treatment modalities

#### Surgery and other local treatments

In papillary microcarcinoma (*i.e.*, papillary tumors ≤1.0 cm) without evidence of extracapsular extension or cervical lymph-node metastases, an initial period of active surveillance can be recommended, albeit with low-quality evidence at present (see below for additional information on active surveillance) (22,33). Otherwise, surgical resection is the most important step in treating patients with DTC. The choice between lobectomy versus total thyroidectomy in low-risk disease is controversial and should be individualized; for intermediate-risk and high-risk disease, total thyroidectomy is the modality of choice (22,33,34,40). Of note, papillary thyroid cancer is multifocal (in some cases bilaterally) in nearly a third of cases (9,10). The role of therapeutic lymph-node dissection is established when lymph nodes are clinically positive, but debated otherwise (22,33,34,40). The most serious complications from thyroidectomy are hypoparathyroidism and (usually unilateral) injury of the recurrent laryngeal nerve. Complications are less common among more experienced surgeons, as well as when lobectomy is performed rather than thyroidectomy (37). Unexpected adverse pathological findings and the local recurrences among patients undergoing lobectomy can usually be addressed by completion thyroidectomy with no expected impact on survival based on retrospective studies (13,22,40). External radiotherapy may be indicated in patients with incomplete tumor resection who are not candidates for completion thyroidectomy, especially when the tumor remnants exhibit low RAI uptake (22,34). However, the recent availability of targeted therapies has restricted the role of external radiotherapy to very selected cases. Finally, there is growing interest in minimally invasive treatment modalities, such as ethanol ablation, cryoablation, or radiofrequency ablation; when available, these modalities may be considered for selected patients with limited thyroid or nodal disease and, increasingly, unresectable locoregional recurrence or limited metastatic disease (22).

#### Active surveillance

A treatment plan that involves close monitoring of the patient without active treatment unless there are changes suggesting disease progression is an established approach in Japan and is under investigation and rapidly acquiring support in Western Countries for very-low-risk papillary thyroid cancer (22,33,40,46). Nevertheless, active surveillance may be an appropriate management strategy for patients with low-risk papillary microcarcinoma (tumor size ≤1 cm) and for very selected patients with tumors measuring >1 cm. Monitoring may include, for example, neck ultrasound every 6 months for 1-2 years and then annually, as well as twice-yearly measurement of thyroid hormones and thyroglobulin, with occasional use of levothyroxine when hypothyroidism is present (22). Active surveillance should not be used when there are aggressive histological types or invasion of adjacent structures or extrathyroidal tissues, in patients who are unable or unwilling to follow-up closely, or by physicians lacking considerable experience in the use of neck ultrasound (22).

#### Thyroid hormones

Since TSH stimulates proliferation of DTC, T4 (in the form of levothyroxine) is used to suppress TSH after surgery) (13). However, the extent of suppression is not yet based on high-level evidence) (22). Levels of TSH < 0.1 mU/L are recommended in high-risk patients, while concentrations between 0.1 mU/L and 0.5 mU/L are recommended in those with intermediate risk) (40). In low-risk disease, thyroglobulin levels may need to be taken into account, with TSH concentrations between 0.5 and 2.0 mU/L recommended for patients with undetectable thyroglobulin and between 0.1 mU/L and 0.5 mU/L otherwise) (40). Patients treated with lobectomy may be able to maintain TSH concentrations between 0.5 mU/L and 2.0 mU/L without levothyroxine replacement) (13,40). Since the subclinical hyperthyroidism induced by hormone therapy can cause or accelerate osteoporosis and negatively affect cardiovascular health, the likelihood and impact of complications must be weighed against the benefit from controlling tumor proliferation (13,22).

### Radioactive iodine treatment and refractoriness

**Figure 1:**
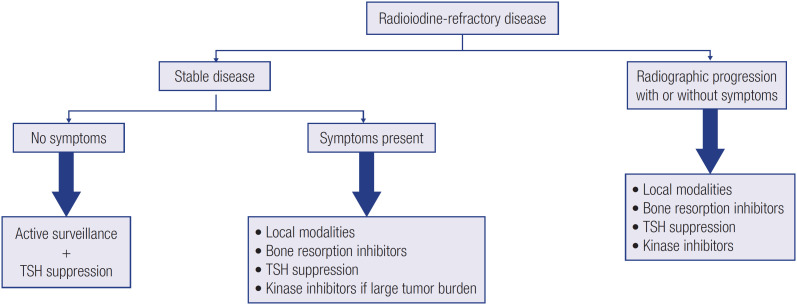
General approach to the patient with radioiodine-refractory disease.

Because thyroid follicular cells transport and incorporate iodide into thyroglobulin, RAI isotopes (such as 123I and 131I) can be used to target these cells in a diagnostic or therapeutic manner. In many cases, DTC cells retain those properties, but some patients have one or more lesions that do not uptake RAI since diagnosis or on follow-up (13). In selected patients, RAI uptake can be enhanced by recombinant TSH administration in the 2 days before RAI therapy or by levothyroxine withdrawal for up to 4-6 weeks before the procedure (34). Therapeutic RAI should be deferred for 6-8 weeks after administration of any iodinated contrast medium (*e.g*., for CT scans), and an iodine-restricted diet should be implemented in the 2 weeks preceding treatment (22,33,34). The procedure is contraindicated during pregnancy, and a negative pregnancy test is advised in women with childbearing potential (34). Although frequently used in the past, diagnostic whole-body scintigraphy has largely been replaced by neck ultrasound and thyroglobulin levels in an attempt to detect persistent disease and to tailor subsequent diagnostic and therapeutic strategies (22,33,40,47). Until recently, postoperative therapeutic RAI was given to most patients with DTC in an adjuvant fashion, with the aim of destroying microscopic foci of neoplastic cells within the thyroid remnant or at distant sites. However, therapeutic RAI has decreased in recent years, and the procedure is no longer recommended in most patients with low-risk disease (22,33,34,40). Even though the evidence regarding this modality in intermediate-risk disease is not compelling, RAI may be useful in a subgroup of these patients, particularly in those with tumors with vascular invasion, unfavorable histologic subtypes and/or high thyroglobulin levels after surgery and persistent structural disease (33,34). Likewise, RAI is indicated in patients with high-risk disease (33,34). Finally, RAI can be used to identify distant metastatic disease, as it may be used simultaneously as a treatment. However, up to a third of patients will have one or more lesions that do not uptake the tracer (33). Of note, RAI refractoriness is defined as either of the following: (1) metastatic disease that does not ever uptake RAI, (2) tumor tissue that loses the ability to uptake RAI after previous evidence of doing so, (3) when RAI is concentrated in some lesions but not in others, or (4) metastatic disease that progresses despite significant RAI uptake (40). There is no indication for RAI treatment in patients classified as refractory to RAI (40). Figure 1 provides a schematic general approach to the patient with RAI-refractory disease.

### Overview of systemic treatment

#### General principles

The greatest recent change in the management of patients with DTC has been the introduction of kinase inhibitors, which play an increasing role in RAI-refractory disease (13,14,20-22,40). In a neoplasm that has been historically characterized by non-responsiveness to conventional chemotherapy, such an introduction has been of paramount importance (18,19). Thyroid cancers are often indolent, even when there are distant metastases (14). Therefore, initiation of systemic treatment can be deferred when an initial period of active surveillance – or even local therapy for structural disease when still feasible, including conventional radiotherapy of bone or brain metastases, as well as resection or stereotactic radiotherapy of brain metastases – confirms that indolent disease is present. When this is not the case, or when indolent disease starts to progress, systemic treatment is indicated. Although not curative, this treatment has been shown to provide significant gains in terms of progression-free survival (PFS). Nevertheless, kinase inhibitors are not devoid of side effects, and these must be considered and adequately managed to ensure the best possible tolerance and quality of life. Of note, bisphosphonates or denosumab can be used in patients with RAI-refractory diffuse or symptomatic bone metastases, either alone or in conjunction with kinase inhibitors (22,33,40).

#### Efficacy of currently approved agents

As shown in Table 2, the currently FDA-approved agents in DTC are cabozantinib, larotrectinib, lenvatinib, pralsetinib, selpercatinib, and sorafenib. Sorafenib and lenvatinib have been the subject of double-blind phase 3 trials comparing these agents against placebo specifically among patients with RAI-refractory DTC (48,49). In the DECISION trial, 417 patients with DTC were randomized 1:1 to sorafenib (starting dose of 400 mg twice a day) or placebo.48 The median PFS was 10.8 months in the sorafenib group and 5.8 months in the placebo group, with a hazard ratio (HR) of 0.59. There was no significant difference in overall survival, but patients in the placebo group with disease progression were allowed to cross over to sorafenib. In the SELECT trial, 392 patients with DTC were randomized 2:1 to lenvatinib (24 mg once daily) or placebo (49). The median PFS was 18.3 months with lenvatinib and 3.6 months in the placebo group, and the HR was 0.21. Once again, crossover was allowed, and there was no significant difference in overall survival in the overall study population. Although these two kinase inhibitors have not been compared head-to-head in a randomized trial, lenvatinib appears to have greater efficacy than sorafenib (14). This can be inferred from a greater effect on PFS, in comparison with placebo, as well as from a greater response rate with lenvatinib (64.8%) than with sorafenib (12.2%) (48,49).

The approval of cabozantinib for patients with previous VEGF receptor-targeted therapy and who are RAI-refractory or ineligible was based on the COSMIC-311 trial (50). In that study, 258 patients with DTC were randomized 2:1 to cabozantinib (60 mg once daily) or placebo, having PFS and the overall response rate as primary endpoints. The median PFS was 11.0 months with cabozantinib and 1.9 months with placebo, and the HR was 0.22 (the other primary endpoint was not met). Larotrectinib was subject to a tumor-agnostic approval based on responses observed in a phase 1/2 trial among patients with *NTRK* fusions (among whom five with thyroid cancer) (51). For pralsetinib, FDA accelerated approval was based on the results of a cohort of nine patients with *RET*-fusion-positive papillary thyroid cancer, eight of whom responding to treatment (52). In the case of selpercatinib, FDA accelerated approval was based on a cohort of 19 patients with *RET*-fusion-positive thyroid cancer (including three with poorly differentiated and two with anaplastic tumors), 15 of whom responding to treatment (53).

Although not specifically approved for this indication, *BRAF* inhibitors (such as dabrafenib/trametinib and vemurafenib) can be considered in selected patients with these alterations after treatment with first-line agents, such as lenvatinib and sorafenib (22).

#### Recognition and management of adverse reactions

Early recognition and management of adverse reactions from kinase inhibitors is critical to enabling patients to benefit from such therapy (Table 2). While some patients experience minimal or no adverse reactions, others will have early or even severe toxicity. Although reliable predictors of toxicity are still absent, higher doses are typically associated with more frequent and intense side effects. Fatigue is probably the most common adverse reaction from kinase inhibitors, which are frequently also associated with hand-foot syndrome, stomatitis, mucositis, and diarrhea. Moreover, because these are antiangiogenic medications, patients may experience new or worsened hypertension and acute cardiovascular complications, such as myocardial infarction and stroke, as well as proteinuria, delayed wound healing, bleeding, perforation, and fistula formation. Table 3 provides an overview of actions to mitigate the frequency or intensity of side effects from kinase inhibitors.

**Table 3:** Actions to help mitigate selected adverse reactions from kinase inhibitors (20)

Adverse reaction	Preventive measures	Therapeutic measures
General recommendation	Patient selection taking into account individual preferences and comorbidities.	Adherence to label recommendations about dose reduction and treatment discontinuation.
Hypertension	Adequate control of blood pressure before treatment initiation; monitoring several times weekly upon treatment initiation, less frequently for 4 to 6 weeks, and then monthly.	Early initiation or change in antihypertensive treatment, with goal of maintaining blood pressure < 140/90 mmHg.
Hand-foot syndrome	Wearing soft, wide shoes and thick cotton socks; avoidance of exposure to hot water, chemicals or sanitizers, and friction or pressure from towels or from physical activities; use of gentle bar soap and moisturizers.	Urea-based creams or topical steroids; referral to a dermatologist and/or podiatrist; use of nonsteroidal anti-inflammatory drugs for pain.
Mucositis, stomatitis	Use of lip balm, alcohol-free mouthwash, and nonabrasive toothpaste; avoidance of salty, acidic, or spicy food.	Compounded mouthwashes (*e.g.*, with tetracycline/dexamethasone/nystatin or sucralfate/magnesium hydroxide and aluminum hydroxide/diphenhydramine).
Skin depigmentation	Use of sunscreen and protective gear; avoidance of direct sunlight.	Not applicable.
Diarrhea	Consider avoidance of fat, dairy products, food with a high fiber content, and coffee; maintain adequate hydration.	Consider loperamide or other antidiarrheal agents.
Wound-healing issues	Interruption of treatment at least 2 weeks before elective surgery.	Topical measures as needed.

In conclusion, much has been accomplished by the thyroid-cancer scientific community, with resulting benefits to patients in terms of a progressive increase in treatment efficacy and safety. Even though decisions about specific diagnostic and therapeutic modalities for patients with DTC are best left to thyroid-specialized endocrinologists, surgeons, nuclear-medicine physicians, and medical oncologists, generalists in these and other disciplines will benefit from increased awareness about the disease. This is particularly important considering the indolent nature of DTC in most patients, which calls for an approach that integrates guideline recommendations into clinical practice in a patient-centric manner.
